# 

**Published:** 1894-04-21

**Authors:** 


**Notice to Advertisers and Subscribers.**

All Advertisements, Orders for Copies of The Hospital, and Business Communications should be addressed to **The Publisher, NOT TO THE EDITOR,** The Hospital, 428, Strand, W.C.

The Cost of Subscription, post free, is as follows:—Per week, 2½d.; per quarter, 2s. 8d.; per half-year, 5s. 3d.; per annum, 10s. 6d. Foreign:—Per Annum, 12s. 8d.; payable, in all cases, in advance.

**Notices of Births, Deaths, and Marriages** are inserted in this column at a charge of 2s. 6d. for an announcement not exceeding four lines.

### NOTICE TO CORRESPONDENTS.

All MS., Letters, Books for Review, and other matters intended for the Editor should be addressed The Editor, The Lodge, Porchester Square, London, W.

The Editor cannot undertake to return rejected MS., even when accompanied by stamped directed envelope.

**"Burdett's Hospital Annual," 1894.**

Fifth year of publication. 900 pp. Boards, gilt lettering. A most complete guide to British, Colonial, Indian, and American Hospitals, Dispensaries, Nursing and Convalescent Institutions, and Asylums. Price 5s. Published by The Scientific Press (Limited), 428, Strand, W.C.

**NOTICE.—The Index for Vol. XIV. (ending Sept. 30th, 1893) is on sale at the Office of this Journal, price 2½d., post free.**

### SCRAPS AND GLEANINGS.

The recently-elected Vice-President of Bengal is a medical man.

The Great Northern Central Hospital has received a further grant of £40 from the Clothworkers' Company.

The Convalescent Home for Railway Servants is doing very excellent work at Mansfield according to its first annual report.

The income from all sources at the Manchester Hospital for Consumption has been £2,966 during the year, the expenditure being £3,200.

The annual report of the Cowes Hospital Ship was of a most satisfactory nature, and no case of infectious disease has been brought into port.

The extension of the Cottage Hospital, Peasley Cross, St. Helens, will be soon completed. The new wards will accommodate 32 beds and six cots.

A valuable present of clothing has again been received at the Launceston Infirmary from Miss Coode for distribution amongst the patients.

The managers of the Asylums Board have adopted plans for the erection of the "Brook" Hospital at Shooter's Hill at an estimated cost of about £195,000.

A concert was again organised this year by the Ladies' Committee in favour of the Children's Hospital Convalescent Fund of the Bradford Children's Hospital.

A concert was given lately at the Brompton Hospital by Madame Dakas, who, with her pupils, on several occasions has helped the committee with providing entertainments.

The report of the past year at the Jessop Hospital for Women, Sheffield, deals with the deficiency of £628, and with an appeal to the subscribers to clear off this deficiency.

Baron Hirsch, who last year distributed among selected charities a sum of £42,000, has this year provided a sum of £15,000 to be divided among London hospitals and charitable institutions.

At the annual meeting of the Huntingdon County Hospital the receipts showed a deficiency of £46, which would have been heavier had not the donations—a most uncertain source of income—been larger than usual.

Lord Armstrong, if he obtains the consent of the High Court to the purchase of Bamborough Castle, Northumberland, intends to endow it with £20,000, and utilize the ancient historic fortress as a convalescent home.

A large bazaar was recently held at Ancoats, Manchester. The object of the bazaar, which was opened by Sir James Fergusson, was to raise the sum of £450 wherewith to endow the Ancoats Cot in the Ancoats Hospital.

The Committee of the Victoria Hospital, Swindon, stated at the general meeting of the institute, which took place recently, that the general receipts, donations, and subscriptions showed a decrease on the year's accounts.

The annual meeting of the Royal Maternity Hospital, Edinburgh, was held this month. The report stated that during the year 285 patients had been admitted to the hospital. The ordinary income had been £799, and the expenditure £701.

Mr. Rider Haggard and Mr. Jerome K. Jerome recently read selections from their works at a drawing-room meeting in aid of the North-Eastern Hospital for Children, Hackney Road, which hospital is in earnest need of funds.

At the annual meeting of the Scottish National Society for the Prevention of Cruelty to Children, held in Glasgow, a report was submitted showing that 1,999 cases, involving the welfare of 4,371 children, were dealt with during the past year.

The Committee of the National Orthopædic Hospital at their last meeting appointed Mr. H. J. Tresidder as secretary. Mr. W. Tresidder, who has for so long occupied the post, will still continue in connection with the work as the hon. secretary.

It is gratifying to us to state that practically the debt of £400 upon the Folkestone Victoria Hospital has been wiped off. The bazaar recently held in favour of the finances of the establishment was a phenomenal success, £374 being raised in one day.

The Committee of the Hinckley Cottage Hospital, at the third annual meeting lately celebrated, showed a falling off in the funds from the ocal warehouse and factory subscriptions. This failure in a most important source of income is a grave loss to the institution.

The Duke of Cambridge presided at St. George's Hospital at a meeting of subscribers to the Dr. Dickenson Testimonial Fund. There are 434 contributors to the testimonial, and £550 has so far been subscribed. The presentation will probably take the form of a portrait and a gift of silver plate.

The Committee of the Sevenoaks Vine Hip Hospital do not report a prosperous year in their annual report. The financial result is a deficit of £49. The six additional beds now available in the hospital have been filled during the greater portion of the year, thus entailing a larger expenditure.

Among numerous offerings which testify to the wide sense of sympathy with the Royal Berkshire Hospital one is particularly conspicuous. It consists of a little avadavat sparrow, who in the midst of recent cold weather flew as an out-patient for refuge to the portico of the hospital. He now sits resplendently caged in the children's ward.

The late Mr. Samuel Weston, of Manchester, has by his will bequeathed £50,000 to the Manchester Royal Infirmary, £10,000 to the Salford Dispensary, £10,000 to St. Mary's Hospital, Manchester, £10,000 to the Convalescent Home, Pendlebury, £5,000 to the Hospital for Incurables, Ardwick, £2,500 to the Manchester Eye Hospital, and £2,500 to the Hospital for Sick Children, Manchester.

Mr. Gibbins, of Ettington, a former liberal donor to the Warneford Hospital, Leamington, has completely fitted up and furnished for the use of that institution a pathological laboratory, with a costly microscope and other suitable instruments. The Warneford trustees, on learning that the cost of the recent extensive draining and sanitary works at the above institution have greatly exceeded the sum expected, generously made a special grant of £125, in addition to their former contributions towards this purpose.

### Medical Vacancies and Appointments.

### VACANCIES.

### Resident Medical Officers.

The following vacancies are announced this week, the amount of salary in each case being given after the name of the institution:

Ancoats Hospital, Manchester.—Resident Junior House Surgeon. Salary £50 per annum, with board and washing. Applications to the Secretary.

City of Liverpool Infectious Diseases Hospital, Parkhill.—Resident Medical Officer, doubly qualified. Salary £100 per annum, increasing £10 yearly to £120, with board, washing, and lodging at the hospital.

Dewsbury and District General Infirmary.—House Surgeon, doubly qualified. Salary commencing at £80 per annum, with board and residence.

Flintshire Dispensary.—House Surgeon. Salary £120 per annum, with furnished house, rent and taxes free; also coal, light, water, and cleaning, or, in lieu thereof, £20 per annum. Knowledge of Welsh desirable.

General Hospital, Birmingham.—Assistant House Surgeon. Appointment for six months. Residence, board, and washing provided. No salary.

Great Northern Central Hospital, Holloway Road, N.—House Physician. Salary £60 per annum, with board and lodging in the hospital.

Royal Victoria Hospital, Bournemouth.—House Surgeon and Secretary. Salary £100 per annum, with board.

West Riding Asylum, Menston, near Leeds.—Resident Clinical Assistant. Appointment for six months. Board and residence provided.

Wirrall Children's Hospital, Woodchurch Road, Birkenhead.—Resident House Surgeon. Salary £50 per annum, with board, lodging on the premises, and washing.

### Non-Resident Medical Officers.

Borough of Eastbourne.—Medical Officer of Health. Salary commencing at £250 per annum, and increasing during satisfactory service by £25 yearly to £350. He will be required to act as Medical Officer to the Sanatorium and as Police Surgeon, for which additional sums of £50 and £25 per annum respectively will be paid. Applications to H. West Forvargue, Town Clerk, Town Hall, Eastbourne, by April 24th.

Dental Hospital of London and London School of Dental Surgery, Leicester Square.—Demonstrator. Honorarium £50 per annum. Applications to Morton Smale, Dean, by May 14th.

Dental Hospital of London, Leicester Square.—Two Assistant Anæsthetists. Applications to J. Francis Pink, Secretary, by May 14th.

St. Marylebone General Dispensary, 77, Welbeck Street, Cavendish Square, W.—Honorary Physician. Applications to the Secretary by April 30th.

### The Hospital Advertiser.

ROYAL VICTORIA HOSPITAL,
BOURNEMOUTH.

